# Remotely Monitoring Change in Vegetation Cover on the Montebello Islands, Western Australia, in Response to Introduced Rodent Eradication

**DOI:** 10.1371/journal.pone.0114095

**Published:** 2014-12-01

**Authors:** Cheryl Lohr, Ricky Van Dongen, Bart Huntley, Lesley Gibson, Keith Morris

**Affiliations:** 1 Department of Parks and Wildlife, Science and Conservation Division, Woodvale, Western Australia, Australia; 2 Department of Parks and Wildlife, GIS Section, Kensington, Western Australia, Australia; 3 Department of Parks and Wildlife, Science and Conservation Division, Keiran McNamara Conservation Science Centre, 17 Dick Perry Drive, Technology Park, Kensington, WA 6151, Australia; University of Queensland, Australia

## Abstract

The Montebello archipelago consists of 218 islands; 80 km from the north-west coast of Western Australia. Before 1912 the islands had a diverse terrestrial fauna. By 1952 several species were locally extinct. Between 1996 and 2011 rodents and cats were eradicated, and 5 mammal and 2 bird species were translocated to the islands. Monitoring of the broader terrestrial ecosystem over time has been limited. We used 20 dry-season Landsat images from 1988 to 2013 and estimation of green fraction cover in nadir photographs taken at 27 sites within the Montebello islands and six sites on Thevenard Island to assess change in vegetation density over time. Analysis of data averaged across the 26-year period suggests that 719 ha out of 2169 ha have increased in vegetation cover by up to 32%, 955 ha have remained stable and 0.6 ha have declined in vegetation cover. Over 492 ha (22%) had no vegetation cover at any time during the period analysed. Chronological clustering analysis identified two breakpoints in the average vegetation cover data occurring in 1997 and 2003, near the beginning and end of the rodent eradication activities. On many islands vegetation cover was declining prior to 1996 but increased after rodents were eradicated from the islands. Data for North West and Trimouille islands were analysed independently because of the potential confounding effect of native fauna being introduced to these islands. Mala (*Lagorchestes hirsutus*) and Shark Bay mice (*Pseudomys fieldi*) both appear to suppress native plant recruitment but not to the same degree as introduced rodents. Future research should assess whether the increase in vegetation cover on the Montebello islands is due to an increase in native or introduced plants.

## Introduction

The Montebello island archipelago consists of 218 islands, islets and rocks situated approximately 80 km from the north-west coast of Western Australia (WA). The largest islands in the archipelago are Hermite (984 ha), Trimouille (498 ha), North West (123 ha), Alpha (103 ha), Bluebell (61 ha) and Renewal (50 ha). Today, the Montebello islands are isolated and uninhabited except for visitation by fishing and tourist charters, recreational vessels and occasional campers.

Prior to 1912, the islands had a diverse terrestrial fauna, which included the golden bandicoot (*Isoodon auratus*), spectacled hare-wallaby (*Lagorchestes conspicillatus*), and spinifexbird (*Eremiornis carteri*) [1]. By 1952, several species had become locally extinct [2] and black rats (*Rattus rattus*), feral cats (*Felis catus*), and house mice (*Mus musculus*) had established [3]–[5]. In 1952 and 1956, three British nuclear weapons tests were completed on the Montebellos and the islands and surrounding seas were placed under Commonwealth control and declared a prohibited area until 1992. In 1992, the Montebellos reverted to WA State control and were declared a Conservation Park.

As part of an agreement between the Commonwealth and the State, introduced rodents and cats were eradicated from the Montebellos [5]. The rodent eradication required five sessions of baiting with brodifacoum, between May 1996 and September 2001, to succeed (Table 1). Feral cats were eradicated by a combination of toxic baiting and trapping. In 1998, 1999, and 2010, five native herbivores and two bird species were translocated to the larger Montebello islands (Table 1) [5]–[7]. Limited monitoring of the broader terrestrial ecosystem followed these management actions.

**Table 1:** Time line of management activities on the Montebello islands since 1995 (5, 6, 7).

Date	Event
August 1995	Trial rodent eradication using ∼100 g Talon-G pellets (0.005% brodifacoum) in bait stations made from plastic bottles on Renewal Island
May–September 1996	11,000 bait stations containing Talon-G installed at 50 m intervals on most islands. 80 islets ‘bombed’ with plastic bags containing Talon-G.
March 1997	Rat sign on Primrose and Crocus islands, islands rebaited.
July 1997	No rat sign on Primrose or Crocus.
June 1998	Thirty mala (*Lagorchestes hirsutus*) introduced to Trimouille Island.
May 1999	Rat sign on Delta, Campbell and Hermite islands. Delta and Campbell rebaited.
June 1999	Shark Bay mouse (*Pseudomys fieldi*) introduced to North West Island.
October 1999	Aerial baiting of Hermite, Renewal, Campbell, Delta, Alpha and Bluebell islands with Pestoff Rodent 20R (0.002% brodifacoum).
August 2000	Rat sign on Hermite.
June 2001	Rat sign on Hermite, Alpha and Bluebell islands.
September 2001	Two rounds aerial baiting 8 days apart on all islands with differential GPS, at 8 kg/ha and 4 kg/ha.
September 2002	No sign of rats.
May 2003	No sign of rats.
January 2010	Barrow Island golden bandicoot (*Isoodon auratus barrowensis*), and spectacled hare-wallaby (*Lagorchestes conspicillatus*) translocated from Barrow Island to Hermite Island.
January 2010	Boodie (*Bettongia lesueur ssp.* WAM M10733) translocated from Barrow Island to Alpha Island.
May 2010	Spinifexbird (*Eremiornis carteri*) and black-and-white fairy wren (*Malurus leucopterus edourdi*) translocated from Barrow Island to Hermite Island.
September 2013	No sign of introduced rodent populations on islands (N. Thomas pers comm.).

Evidence that introduced rodents alter terrestrial ecosystems is rare or difficult to quantify. Some studies have documented the direct and indirect effects of introduced rodents on native fauna and flora [8]. In many cases, evidence of changes to vegetation structure or composition due to the presence or removal of rodents is confounded by multi-species eradications [9],[10], other disturbance events [11], or only observed in relation to changes in faunal populations [12],[13]. Two studies revealed that introduced rodents can alter the composition of coastal forests and Californian grasslands, primarily through seed consumption and reduced plant recruitment [14],[15].

Recent developments in remote sensing technologies, and the characteristics of the Montebello islands, presented an opportunity to measure long-term changes in a terrestrial island ecosystem before and after a rodent eradication and native fauna translocations. The geology, isolation from human disturbance and arid-tropical climate of the islands has resulted in a relatively simple terrestrial ecosystem. Today, the islands are reserved for conservation and experience minimal ecological disturbance. Additionally, the large number of islands in the archipelago provides a study region with many similar, but relatively independent sites for analysis.

## Methods

### Vegetation cover estimation

Vegetation cover on the Montebello island archipelago was estimated via regression of green fraction cover in nadir photographs taken in 2013 against Landsat imagery collected between 1988 and 2013 (Supporting Information S1). Field measures of cover were collected at 27 sites on four islands within the Montebello islands (−20.435°S; 115.549°E) and six sites on Thevenard Island (−21.456°S; 115.000°E), an island located 125 km SSW of the Montebellos, in September and December 2013, respectively, using a method of nadir digital photography [16]. Sites were chosen to represent homogeneous vegetation at different cover values. Thevenard Island provided additional sites with low vegetation cover. At each site, a 90 by 90 m plot, which covers a grid of nine (30 by 30 m) Landsat pixels, was added. Within each plot, nine points set out in a regular grid were sampled. These sample points were spread throughout a 50 by 50 m sub-plot which was located in the centre of the larger 90 by 90 m plot. At each sample point, four nadir digital photographs were taken from a height of 4.2 m using a telescopic pole at each of the cardinal directions. No specific permissions were required for the collection of this data. This research did not involve interaction with endangered or protected species.

Automatic estimation of the green fraction cover in the nadir photographs was undertaken using a program written for Matlab [16]. The program accounts for variations in illumination and then classifies the image. The red, green and blue digital numbers from the original images are converted using a Green Leaf Algorithm (GLA) and to CIE L*a*b* colour model values before further analysis.

### Dry Season Landsat Imagery Analysis

**Figure 1:**
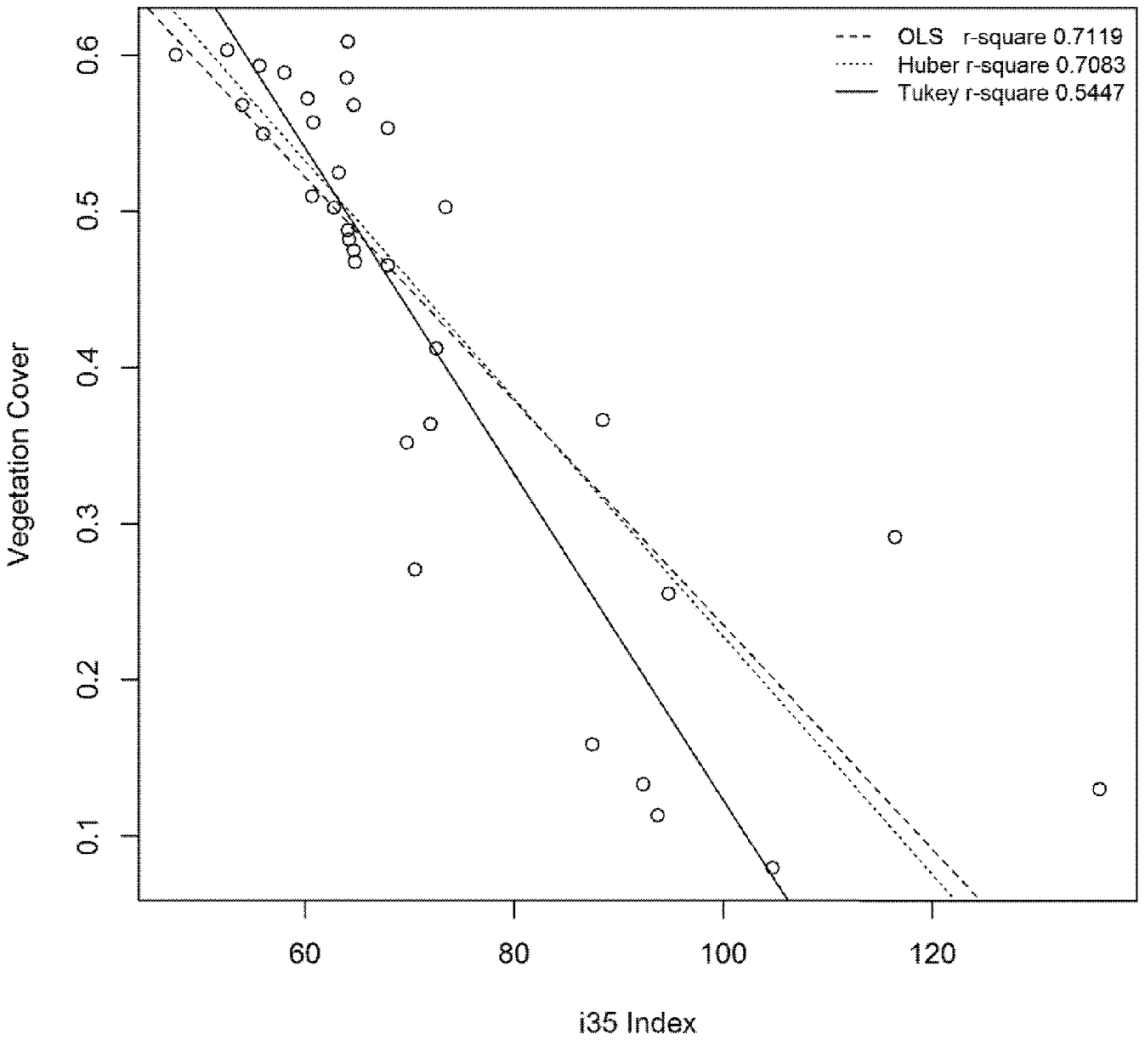
Regression equations used to calibrate i35 vegetation index with field measures of vegetation cover on 4 Montebello islands and Thevenard Island.

Twenty six dry-season (November/December) Landsat images of the Montebello islands from 1988 to 2013 were downloaded from the United States Geological Survey website [17]. Landsat data is captured across seven spectral bands. These bands can be combined to form vegetation indices [18]. The i35 ((band 3 + band 5)/2) vegetation index is commonly used in Western Australia to map and monitor vegetation cover and condition [19]–[21]. Here, the i35 index was applied to Landsat 8 Operational Land Imager (OLI) images captured on 18 September 2013 (Montebellos) and 14 December 2013 (Thevenard). These image capture dates were selected to align as closely as possible to the time of the field measurements. The index was then regressed against vegetation cover estimates from the field using an ordinary least squares method (r^2^ = 0.71; Figure 1) [22], using the R software environment (R Core Team 2012). This robust form of regression minimises the sum of squared vertical distances from the observation and the predicted values. Large residual errors at low cover values are likely due to the small contribution of green vegetation cover to overall reflectance at these sites. When vegetation cover is low, reflectance is dominated by the proportions of litter, exposed soil and rock. Soil colour is also likely to be a factor. However, in this study, these variables have not been accounted for. The modelled relationship between satellite imagery and vegetation cover was applied to the sequence of 26 Landsat images, producing cover images for each year.

The cover images produced were subject to trend analysis [20],[23] in which images are placed in an image stack and the average cover value per field plot, and per island, were plotted through time (Supporting Information S2). Change in cover was also summarised spatially by calculating the linear trend in time for each pixel. Equation 1 was applied to the image stack using the image processing software ERDAS ER Mapper v11, with β equalling the calculated trend through time for each pixel in an image stack, *t* the years 1987 to 2012 and *Y_t_* the pixel value at year *t*.
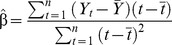
(Equation 1)


**Figure 2:**
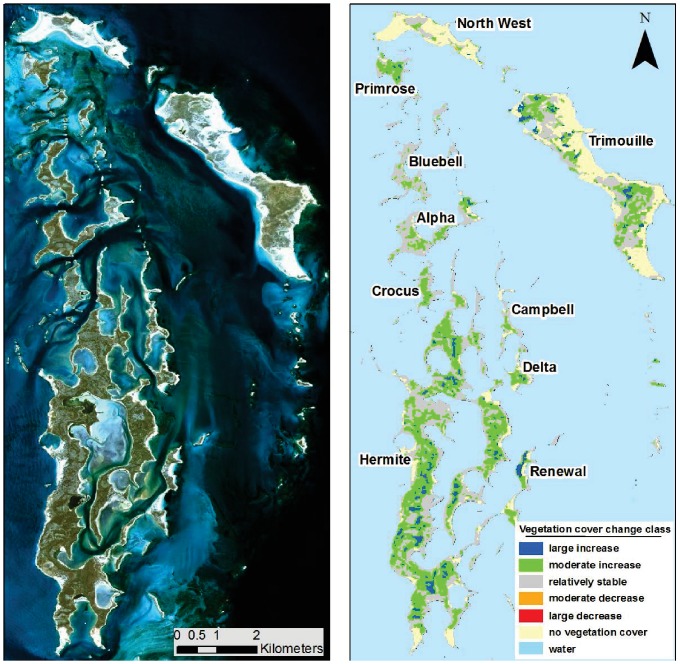
Foliage cover trend map of the Montebello island archipelago for years 1987–2012.

The resulting trend image provides a visual representation of areas where vegetation cover is increasing or decreasing (Figure 2). The trend image was classified into areas with change of a similar degree. The class boundaries, and geographic areas attributed to each class, are shown in Table 2.

**Table 2:** Trends and definitions of the vegetation cover index on the Montebello archipelago.

Vegetation cover trends	Average annual change in vegetation cover over 26 year period	Area (ha)	% Montebello archipelago
Large decrease in vegetation cover	Loss >−1% per year	0	0
Moderate decrease in vegetation cover	loss −1% to −0.5% per year	0.64	0.03
Relatively stable	Δ −0.5% to 0.5% per year	955.89	44.07
Moderate increase in vegetation cover	gain 0.5% to 1% per year	637.39	29.39
Large increase in vegetation cover	gain> 1% per year	82.13	3.79
No vegetation cover during the 26 year time period	-	492.95	22.73

### Time Series Analysis of Landsat Imagery

The time series vegetation cover across the Montebellos was analysed via chronological clustering using the software Brodgar 2.7.1 (www.highstat.com/brodgar). Chronological clustering analysis identifies successional steps in ecological data by identifying successional groups of data points (n>1) that are significantly different from other groups of data points occurring immediately before or after the group of interest [24]. Significantly different singletons (n = 1) are identified as outliers. “Brodgar” generates results at several α-levels because continuous ecological succession or a single cluster of data points, rather than separate successional steps, is more likely to be identified at lower α-levels. Larger α-levels allow the identification of less significant ecological events that may be used to define successional steps [24] (Supporting Information S3).

The average annual vegetation cover for seven of the larger Montebello islands was analysed using Brodgar, including Hermite, Delta, Renewal, Campbell, Alpha, Bluebell, and Primrose. Hermite Island, being very large was further divided into three separate sites (Central Hermite, East Hermite and North Hermite), creating nine sites for analysis via chronological clustering (Supporting Information S2). The results of the chronological clustering analysis were used to define statistically significant break-points in the vegetation cover index and hence subsets of potentially ecologically significant time-series data for further analysis (Supporting Information S3).

To identify the biological events that may be creating break-points, the vegetation cover values, rainfall data from the monitoring station at Dampier (which is approximately 120 km from the Montebello islands) [25], field site, and year for each subset of data were further analysed via linear mixed-effects models in RStudio© 2009–2012 version 0.97.551. Data for Trimouille and North West islands were also analysed independently of others as the mala (*Lagorchestes hirsutus*) and Shark Bay mouse (*Pseudomys fieldi*) were introduced to those islands between 1998 and 2000 (Table 1).

## Results

### Trends in vegetation cover

**Figure 3:**
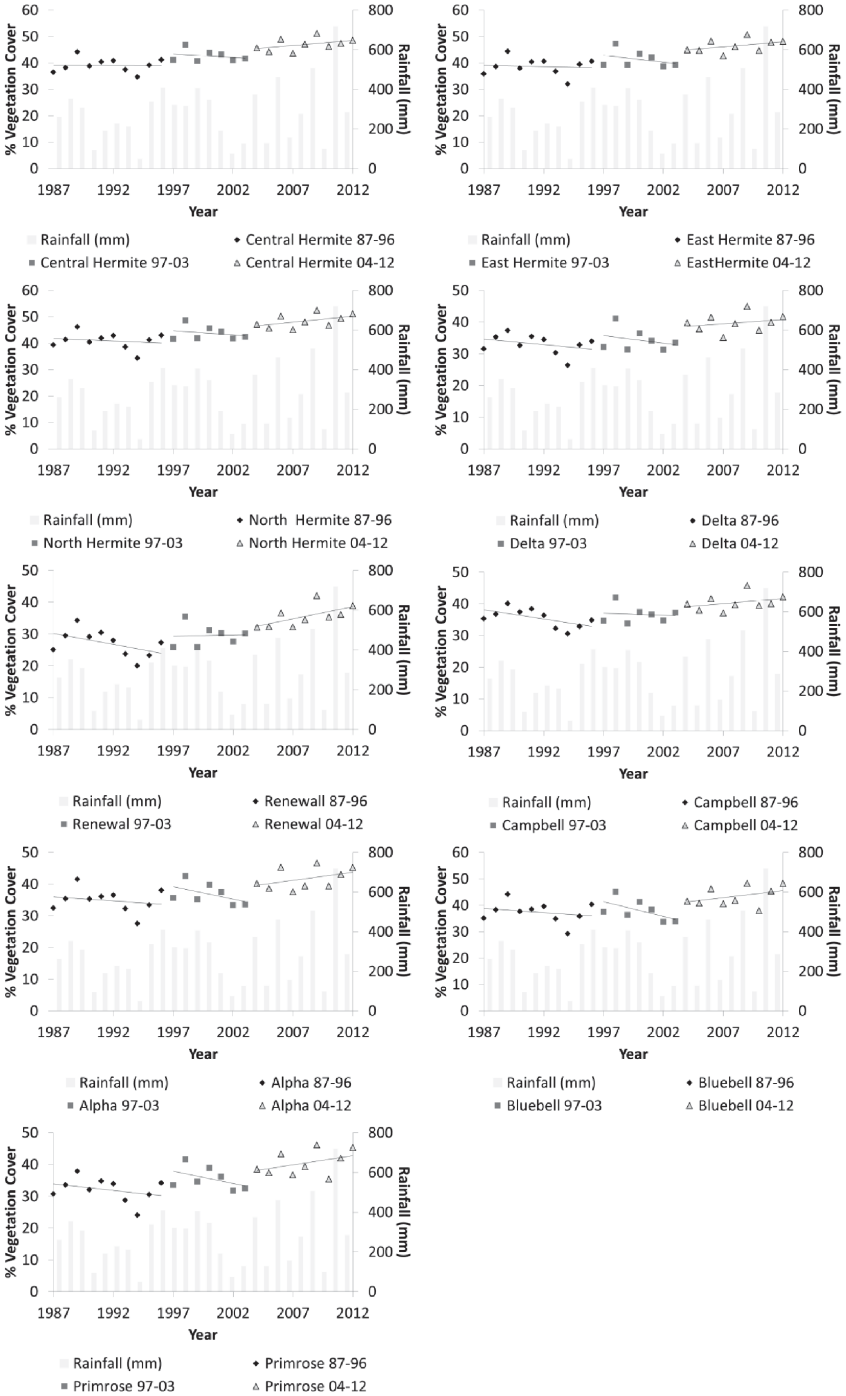
Percentage vegetation cover on nine sites in the Montebello archipelago with linear regression shown for each time period and rainfall between 1987 and 2012.

**Figure 4:**
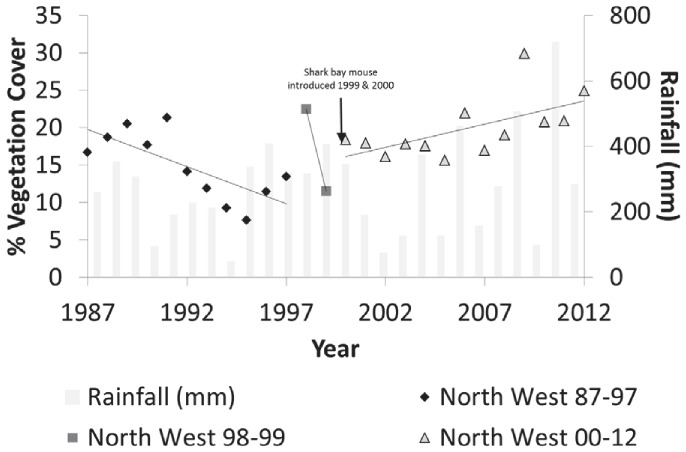
Percentage vegetation cover on North West Island with linear regression for each time period and rainfall between 1987 and 2012.

Results suggest that 719 ha out of 2169 ha (33% of the Montebello archipelago) have increased in vegetation cover by up to 32%, 955 ha (44%) have remained stable and 0.6 ha have declined in vegetation cover. Over 492 ha (22%) had no vegetation cover at any time during the 26 year period analysed (Table 2; Figure 2). Renewal Island experienced the greatest increase in vegetation cover, changing from a minimum of 20% cover in 1994 to a maximum of 42% cover in 2009 (Figure 3). North West Island experienced the least increase in vegetation cover, changing from a minimum of 7%in 1995 to a maximum of 29% in 2009 (Figure 4).

Chronological clustering analysis suggests two primary breakpoints in the average vegetation cover data occurring at 1997 and 2003. Hermite, East Hermite, North Hermite, Delta, Campbell, Bluebell, and Primrose all experienced a break-point in vegetation cover data in 2003, whereas only Campbell, Renewal and North West islands experienced a break-point in 1997. Secondary break-points also appear in 1992 and 2008. This analysis independently identified statistically significant break-points which are offset from the beginning and end of rodent eradication activities by one to two years.

Prior to the rodent eradication between 1987 and 1996, rainfall (p = 0.19) was not a significant explanatory variables for change in vegetation cover (Table 3); whereas during and after the eradications, both year (p<0.001) and rainfall (p<<0.001) were significant variables explaining variation in vegetation cover. Between 1997 and 2003, rainfall declined significantly over time (Adjusted r^2^ = 0.56, F_1,5_ = 8.65, p = 0.03). Vegetation cover did not decline across most of the islands despite declining rainfall levels during this period. On many islands vegetation cover had been declining prior to the rodent eradication (1987–1996) but increased after rodents were successfully eradicated from the islands (2004–2012; Figure 3).

**Table 3:** Parameter estimates for the independent variables year and rainfall in each of the time periods identified via chronological clustering analysis.

Period	Year	Rainfall
Prior eradication: 1987–1996	−0.015***	−0.001
During eradication: 1997–2003	−0.028**	−0.021***
After eradication: 2004–2012	0.026***	0.007***

Level of significance: ***<0.001; **0.001; *0.01.

**Figure 5:**
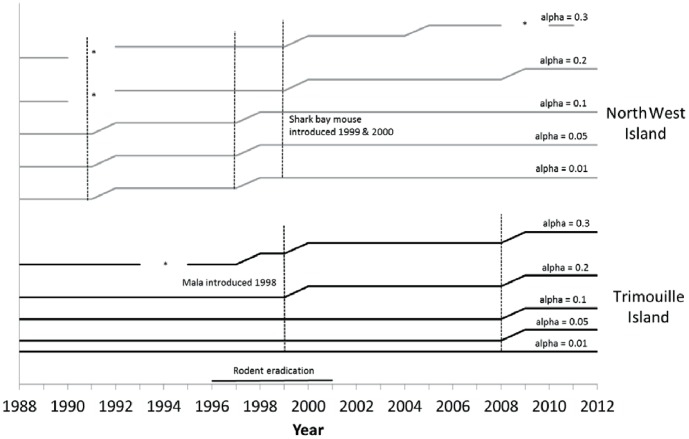
Chronological clustering of vegetation data for North West and Trimouille Island between 1987 and 2012.

**Figure 6:**
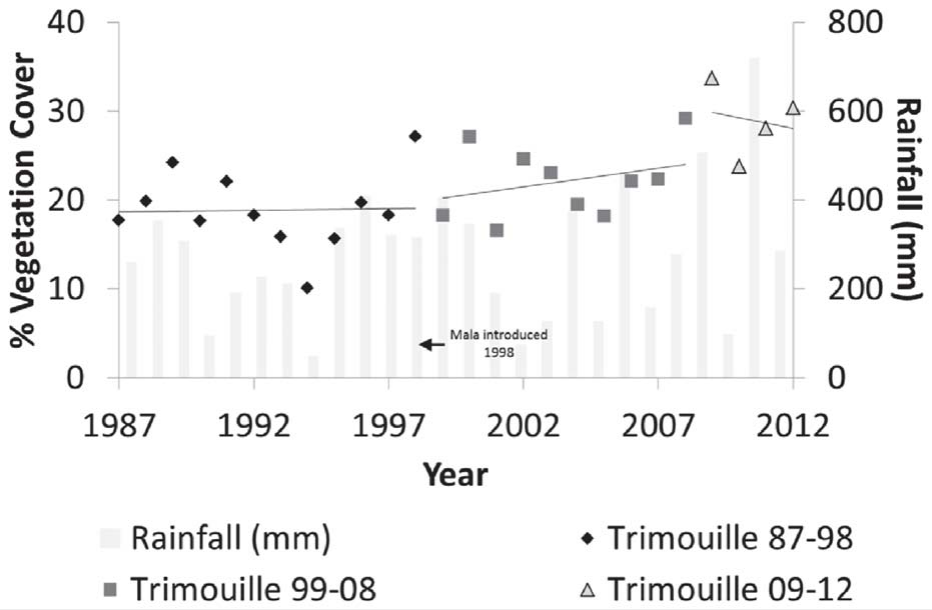
Percentage vegetation cover on Trimouille Island with linear regression shown for each time period and rainfall between 1987 and 2012.

Data for North West and Trimouille islands were analysed independently because of the potential confounding effect of native fauna being introduced to the islands between 1998 and 2000. Break-points for Trimouille occurred primarily at 2008 and secondarily at 1999, shortly after mala were introduced to the island (Figure 5). Before the rodent eradication and mala introduction, vegetation cover remained relatively stable at approximately 6% cover. After the rodent eradication and mala introduction, vegetation cover increased by approximately 8% (Figure 6), which is half the average 16% increase in vegetation cover recorded for other islands between 2003 and 2012. Neither rainfall nor year were significant explanatory variables for change in vegetation cover on Trimouille either before or after the rodent eradication.

Primary break-points occurred on North West Island in 1997 and 1999, one year after the first rodent eradication attempt and when the Shark Bay mouse was introduced to the island (Figure 5). The short time frame between these breaks suggests a single transition period that occurred over a couple of years. A secondary break-point occurred in 1991. The event in 1991 became an aberrant data point at larger alpha-levels. Prior to the rodent eradication and the introduction of the Shark Bay mouse (1987–1997) on North West Island, vegetation cover declined slightly, but rainfall was not a significant explanatory variable (F_1,7_ = 0.006; p = 0.93), unlike year (F_1,7_ = 8.95, p = 0.02; Table 3; Figure 4). After introduced rodents were eradicated and the Shark Bay mouse introduced (2000–2012), vegetation cover increased by 9.5% and rainfall became a significant explanatory variable (F_1,9_ = 5.43, p = 0.04), but not year (F_1,9_ = 3.51; p = 0.09).

## Discussion

The results presented here suggest that introduced rodents can influence successional change in vegetation cover on islands. The chronological clustering technique we used [24] is specifically designed to identify groups of data that indicate successional change within an ecological parameter. Further analysis via traditional statistical methods like linear models can then be used to identify potential causes of change. Our analysis independently identified break-points in the vegetation cover data for the Montebello islands that coincided with known management activities (albeit with some time-lag). On Renewal Island there was evidence of declining vegetation cover in the presence of introduced rodents. Black rats are known to attack vegetation during all life-stages: they are seed predators that prevent recruitment [15],[26],[27], and grazers or browsers [28] that can prevent reproduction or cause the indirect mortality of plants through ring-barking [29]. The impact of house mice on vegetation is less obvious, although like rats, mice can consume enough grass seed to alter grassland species composition [14]. We also show an increasing trend in vegetation cover following the successful eradication of introduced rodents from the Montebello islands. The absence of a positive relationship between rainfall and vegetation cover during the eradication phase further suggests that the observed decline in vegetation cover was likely to be related to the presence of rodents. When rodents had been absent for 2 years (i.e. in 2003) a significant positive relationship between rainfall and vegetation cover became apparent, indicating rodents were no longer suppressing vegetation growth.

On North West Island, introduced rodents were replaced with the native Shark Bay mouse [6]. Despite the introduction of the native rodent, the vegetation cover on this island increased. This observation suggests that the Shark Bay mouse may not suppress native plant recruitment to the same degree as introduced rodents. Little is known about the diet of the Shark Bay mouse other than it has a diverse diet consisting of seeds, fungi, flowers, and invertebrates [6],[30],[31]. Similarly, little is known about the ecology of the Shark Bay mouse. Interestingly, one study found that the trap success rate of Shark Bay mice on Bernier Island, Western Australia, was positively correlated with the percentage cover of *Spinifex longifolius*, a native plant common on the Montebello islands (Speldewinde and Morris unpublished data).

While vegetation cover on Trimouille Island responded positively to rodent eradication, this response was not as rapid as on other islands. In this case, the introduction of the mala to this island shortly after the first rodent eradication attempt potentially slowed the recovery of the vegetation. Mala are herbivores that principally consume perennial grasses and monocot seeds, and secondarily shrubs [32],[33].

One question raised by our study is whether the increase in vegetation cover is due to an increase in native or introduced plants. Some of our field sites which were dominated by spinifex in 2013 had experienced an increase in vegetation cover over the years covered by this study. But at least six introduced plant species have also been observed on the Montebello islands, including kapok (*Aerva javanica*) and buffel grass (*Cenchrus ciliaris*), which are both widespread. (M. Lohr unpublished data). The earliest record of kapok on the Montebello islands is from 1990 [34]. This plant is now widespread across at least four islands: Hermite, Renewal, Crocus and Alpha. Buffel grass is also a successful invader, especially on disturbed or modified landscapes [35]. Additionally, introduced rodents, particularly black rats, are capable of facilitating the spread of invasive plant species [36]. Identification of the plant species contributing to the changes in vegetation cover is important in the context of the conservation status of the islands, and should be the focus of further studies. Further introductions of native fauna species to the Montebello islands in 2010 [specifically boodies (*Bettongia lesueur*), golden bandicoots (*Isoodon auratus*) and hare-wallabies (*Lagorchestes conspicillatus*)] also provide future opportunities for examining the influence of these species on island vegetation.

## Supporting Information

Supporting Information S1 **Estimates of vegetation cover for satellite imagery calibration.**
(XLSX)

Supporting Information S2 **Annual vegetation cover estimates for Montebello islands.**
(XLSX)

Supporting Information S3 **Chronological clustering results for Montebello islands.**
(XLSX)
